# Long-Term Response and Survival With Liposomal Irinotecan and Fluorouracil in a Patient With Locally Advanced Pancreatic Ductal Adenocarcinoma: A Case Report

**DOI:** 10.7759/cureus.113923

**Published:** 2026-08-04

**Authors:** Skaiste Tulyte-Kirzova, Edvina Vitkauskaite, Liutauras Gumbys

**Affiliations:** 1 Faculty of Medicine, Vilnius University, Vilnius, LTU; 2 Hematology, Oncology, and Transfusion Medicine Center, Vilnius University Hospital Santaros Klinikos, Vilnius, LTU; 3 Radiology and Nuclear Medicine, Vilnius University Hospital Santaros Klinikos, Vilnius, LTU

**Keywords:** case report, liposomal irinotecan, long-term survival, pancreatic ductal adenocarcinoma, third-line

## Abstract

Pancreatic ductal adenocarcinoma (PDAC) is frequently diagnosed in the advanced stages. Despite available first-line systemic chemotherapies, prognosis for patients with PDAC remains poor. The combination of liposomal irinotecan plus fluorouracil (5-FU) and leucovorin (LV) is approved for the treatment of patients with metastatic PDAC who have previously progressed on gemcitabine-based chemotherapy.

This case study reports on a 56-year-old female who was diagnosed with locally advanced stage III PDAC (T4, N2, M0). The tumor was judged inoperable by the multidisciplinary team (MDT), and the decision was taken to initiate systemic therapy. First-line modified FOLFIRINOX (fluorouracil (5-FU), leucovorin (LV), irinotecan, and oxaliplatin) was administered for 12 cycles, as per local standards, over six months and achieved a partial response (PR). Second-line gemcitabine monotherapy was initiated five months later (11 months after diagnosis) after computed tomographic (CT) imaging revealed new lesions in the interlobar pleura of the left lung. A best response of stable disease was achieved. Following disease progression, third-line liposomal irinotecan plus 5-FU was started 10 months after second-line treatment initiation (and 21 months after diagnosis). Best response was PR. Treatment continued until evidence of new lesions 35 months later (and 56 months after diagnosis). The patient was subsequently switched to fourth-line cisplatin/gemcitabine. All treatments were generally well tolerated. The patient demonstrated a good performance status throughout treatment.

Remarkable findings of a sustained response for almost three years with third-line liposomal irinotecan plus 5-FU in our patient with locally advanced disease offer the potential for improving the current poor prognosis associated with PDAC.

## Introduction

Pancreatic ductal adenocarcinoma (PDAC) is the most frequent pancreatic cancer subtype (>85% of cases) [1,2]. New cases and deaths attributed to PDAC are steadily increasing in Europe, and PDAC mortality is projected to rank second only to lung cancer as the leading cause of cancer-related deaths overall by 2040 [2,3]. PDAC is frequently diagnosed at an advanced stage, when the disease has spread locally or when metastases have developed, and this confers a poor prognosis [3,4]. Systemic chemotherapy based on gemcitabine alone or in combination (with nab-paclitaxel or other chemotherapies) or FOLFIRINOX (5-fluorouracil (5-FU), leucovorin (LV), irinotecan, and oxaliplatin) is recommended as first-line treatment for patients with non-resectable PDAC [5,6]. Despite proven survival benefits in the first-line setting, efficacy of later-line treatment remains an area of unmet need for patients with PDAC. Liposomal irinotecan (Onivyde®; Servier, Paris, France), a topoisomerase I inhibitor, is an intravenous formulation of irinotecan encapsulated within a liposome that is approved in combination with 5-FU and LV for the treatment of patients with metastatic PDAC following progression on gemcitabine-based chemotherapy. Liposomal irinotecan plus 5-FU/LV demonstrated improved overall survival (OS) versus 5-FU and LV alone (6.1 months and 4.2 months, respectively) in patients with metastatic PDAC previously treated with gemcitabine-based therapy in the global, phase 3, open-label, randomized NAPOLI-1 trial [7,8]. Accordingly, liposomal irinotecan plus 5-FU/LV is recommended by international guidelines for the treatment of patients with advanced pancreatic cancer previously treated with a gemcitabine-based regimen [5,6].

In this report, we present the case of a female patient diagnosed with locally advanced PDAC, treated with first-line mFOLFIRINOX chemotherapy, who achieved a subsequent prolonged response and survival with liposomal irinotecan plus 5-FU/LV in the third-line setting.

## Case presentation

**Figure 1:**
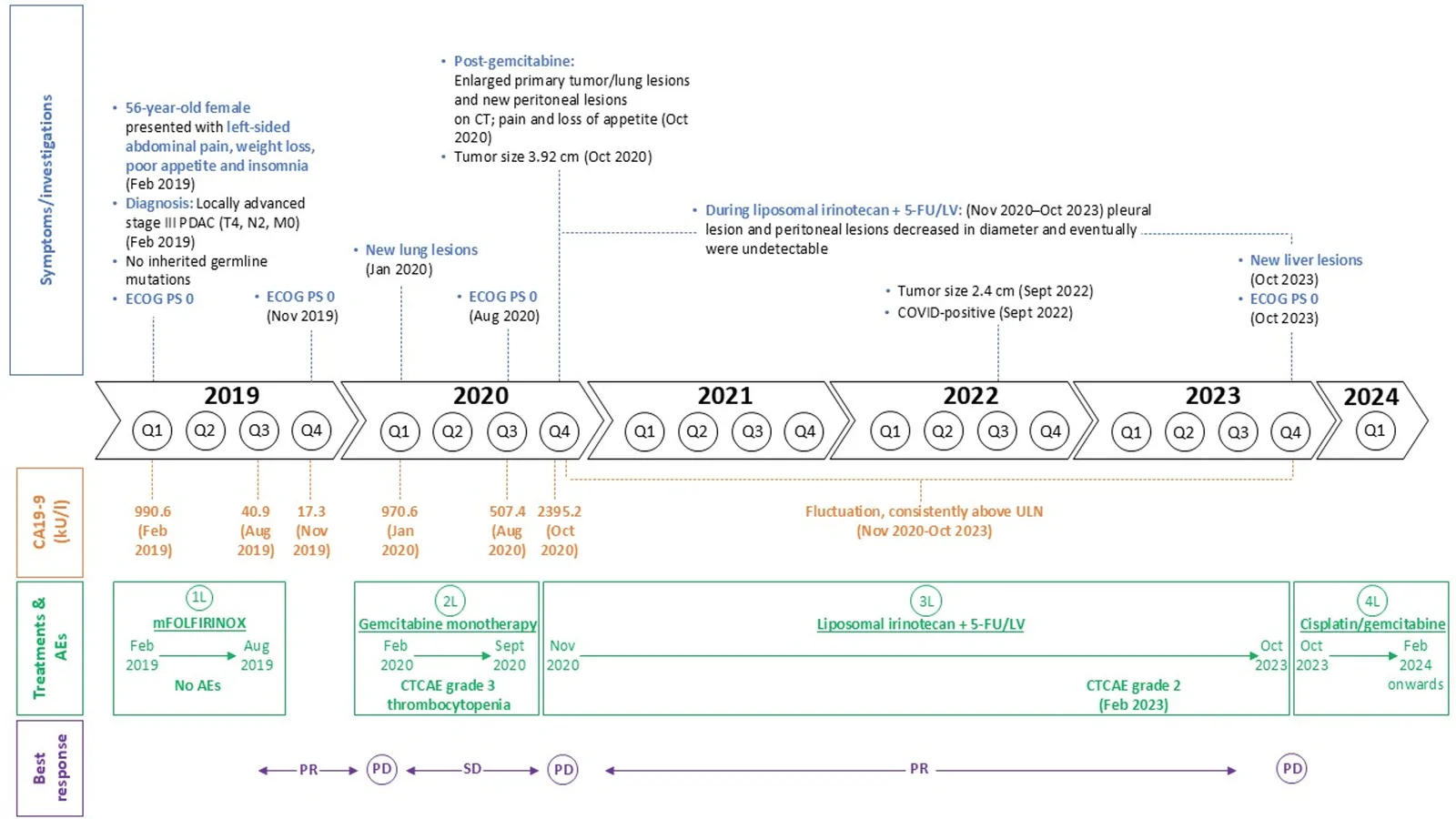
Case study timeline 1L, first line; 2L, second line; 3L, third line; 4L, fourth line; AE, adverse event; CA 19-9, carbohydrate antigen 19-9; CT, computed tomography; CTCAE, Common Terminology Criteria for Adverse Events; ECOG PS, Eastern Cooperative Oncology Group performance status; PD, progressive disease; PDAC, pancreatic adenocarcinoma; PR, partial response; Q, quarter; SD, stable disease; ULN, upper limit of normal 37 kU/L for CA 19-9.

**Figure 2:**
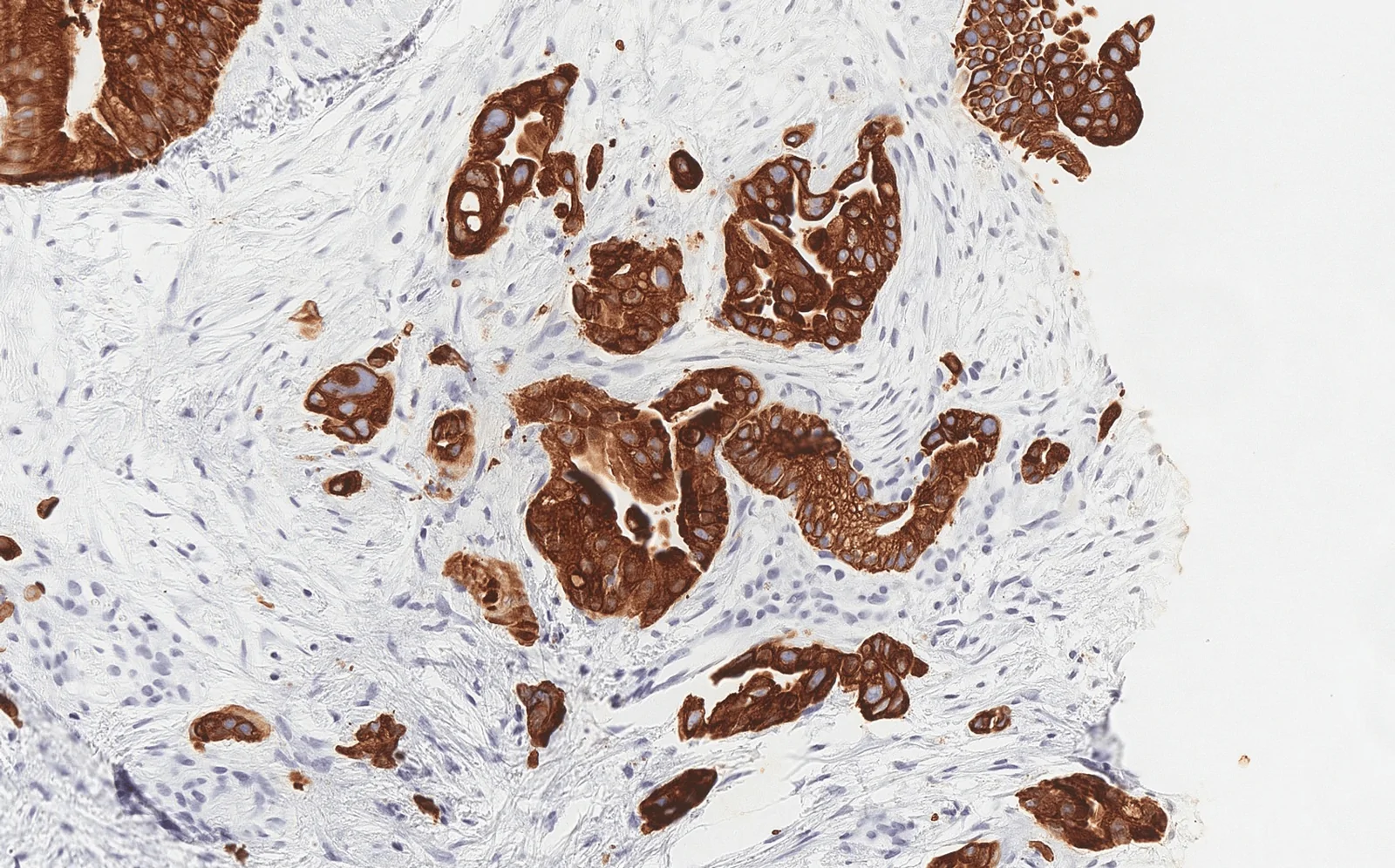
Pathology findings at diagnosis Immunohistochemical findings of the ductal adenocarcinoma; tumor cells were strongly positive for cytokeratin 7 (original magnification 20×).

**Figure 3:**
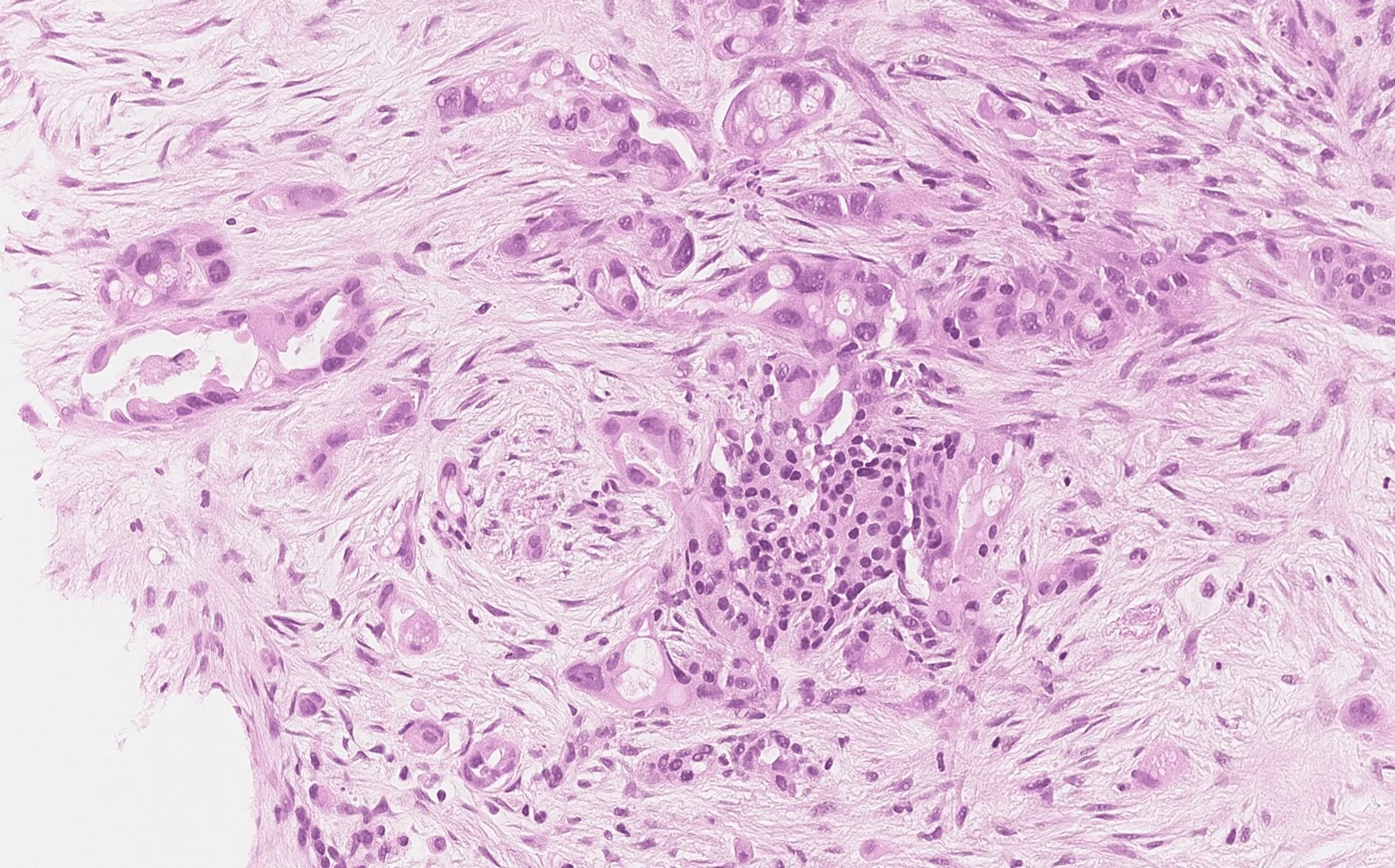
Pathology findings at diagnosis Microscopical aspects of ductal adenocarcinoma (hematoxylin and eosin (H&E) stain): infiltrating irregularly shaped and variably sized glandular and ductal structures with marked nuclear pleomorphism (original magnification 20×).

**Figure 4:**
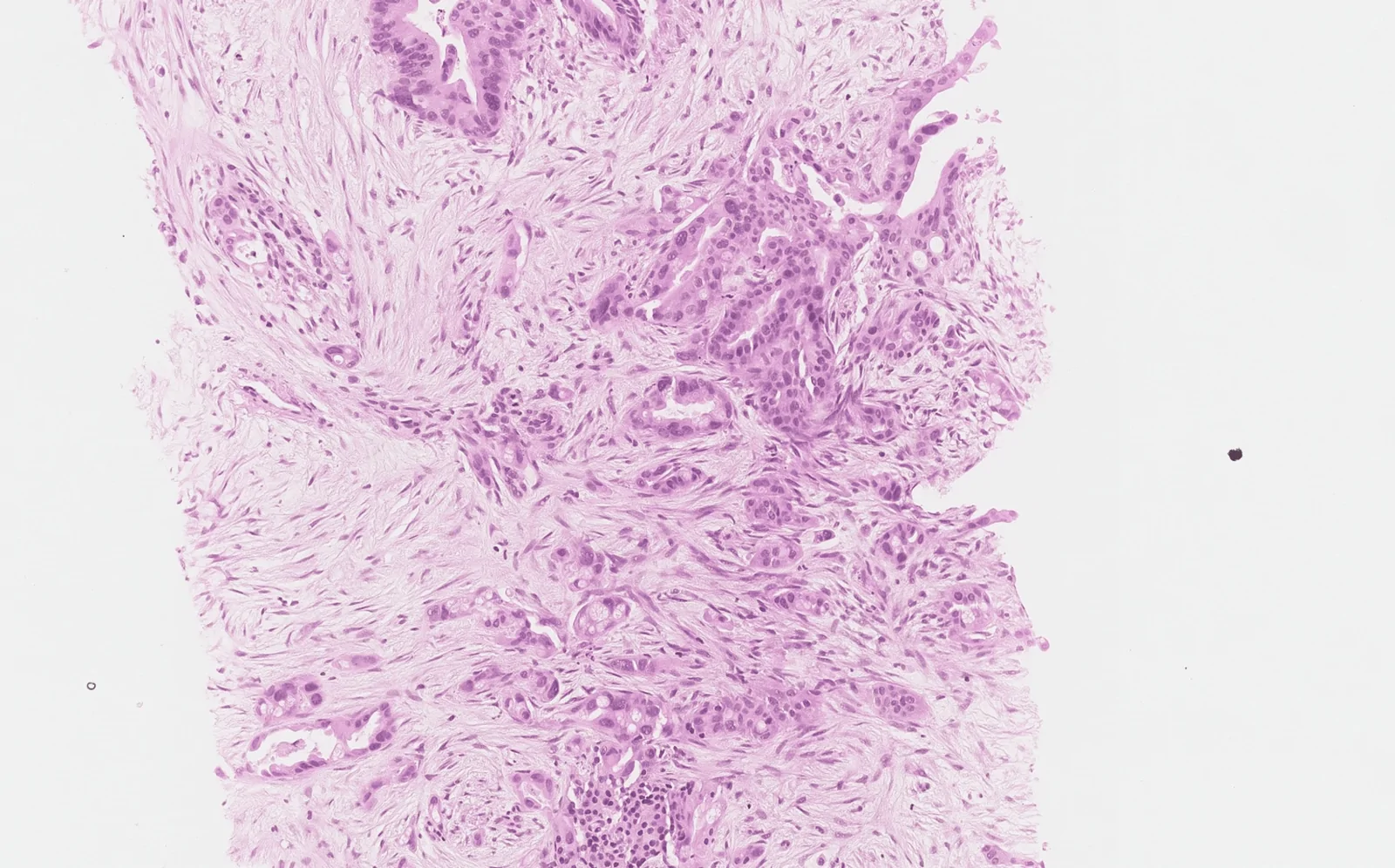
Pathology findings at diagnosis Microscopic aspects of ductal adenocarcinoma (hematoxylin and eosin (H&E) stain): infiltrating irregularly shaped and variably sized glandular and ductal structures surrounded by remarkably desmoplastic stroma (original magnification 10×).

A 56-year-old Caucasian female presented to the Vilnius University Hospital clinic (Vilnius, Lithuania) with abdominal pain on her left side, weight loss, poor appetite, and insomnia (see case summary in Figure 1). The patient had a history of type 2 diabetes (for the previous six months), which had been controlled with metformin 500 mg daily. She was also receiving nebivolol 5 mg daily for primary arterial hypertension. Ultrasound (US) performed at our hospital revealed a pancreatic mass measuring 6.5 cm in diameter with vascular invasion. Subsequent diagnostic work-up comprised contrast-enhanced, high-resolution, computed tomography (CT) imaging three days after presentation and an US-guided biopsy four days after presentation. Pathology results identified moderately differentiated grade 2 adenocarcinoma of the pancreas (Figures 2-4). Furthermore, CT confirmed a tumor located in the pancreatic body and tail, with imaging results showing invasion of the vascular space (coeliac trunk and splenic vein) and para-aortic lymphadenopathy. Collectively, these results confirmed a diagnosis of locally advanced stage III PDAC (T4, N2, M0). At diagnosis, the patient had a good functional status, as demonstrated by an Eastern Cooperative Oncology Performance Status (ECOG PS) score of 0. Laboratory evaluation showed an increase in the tumor marker carbohydrate antigen 19-9 (CA 19-9; 990.6 kU/L; upper limit of normal (ULN), 37 kU/L), and next-generation sequencing revealed a molecular profile with no inherited germline mutations in the *APC*, *BMPR1A*, *BRCA1*, *BRCA2*, *CDKN2A*, *MEN1*, *MLH1*, *MSH2*, *MSH6*, *PMS2*, *PALB2*, *SMAD4*, *STK11*, *TP53*, *TSC1*, *TSC2*, and *VHL* genes. Biochemistry and organ function testing showed no clinically significant findings.

After discussion of the findings, the multidisciplinary team (MDT) reached a decision to treat the patient with systemic therapy for inoperable disease (due to invasion of the vascular space and, in particular, the para-aortic lymphadenopathy). As a result, first-line chemotherapy with mFOLFIRINOX was initiated the same month and continued for 12 cycles (as per local guidance), over six months with no dose reductions or delays. mFOLFIRINOX was chosen for first-line treatment as the patient had a favorable performance status, indicating that she would tolerate a combination therapy. CT tumor evaluation showed a best response of partial response (PR) (according to RECIST 1.1), with an approximately 29% reduction in the longest diameter of the primary tumor. Moreover, serum CA 19-9 levels were shown to have declined from 990.6 kU/L at diagnosis to just above the ULN (37 kU/L), reaching 40.9 kU/L at the end of mFOLFIRINOX chemotherapy, and continued to decrease until three months post-chemotherapy (17.3 kU/L). During first-line treatment, the patient did not report significant adverse events (AEs), but granulocyte colony-stimulating factor (G-CSF) was administered for neutropenia prophylaxis. ECOG PS remained 0. Upon re-examination at the end of first-line treatment, the MDT maintained their decision that the tumor was inoperable (due to continued vascular involvement); therefore, the patient underwent routine CT assessment.

Five months after first-line treatment ended (11 months after diagnosis), CT revealed progression with new lesions in the interlobar pleura of the left lung, but the primary tumor remained stable. Although CA 19-9 levels had increased to 970.6 kU/L (ULN 37 kU/L) at this time point, the patient remained asymptomatic. One month later (12 months after diagnosis), the patient initiated second-line gemcitabine monotherapy at 1000 mg/kg on days 1, 8, and 15 of every 28-day cycle. During treatment, the patient experienced an AE of grade 3 thrombocytopenia classified according to Common Terminology Criteria for Adverse Events (CTCAE; version 5.0), which led to a 25% dose reduction of gemcitabine. The patient received gemcitabine monotherapy for six months and achieved a best response of stable disease, with ECOG PS remaining 0. While the pleural lesion was enlarged by 13% at the end of gemcitabine treatment, no new lesions were identified, and the patient remained asymptomatic. Additionally, serum CA 19-9 levels had decreased to 507.4 kU/L (ULN 37 kU/L).

**Figure 5:**
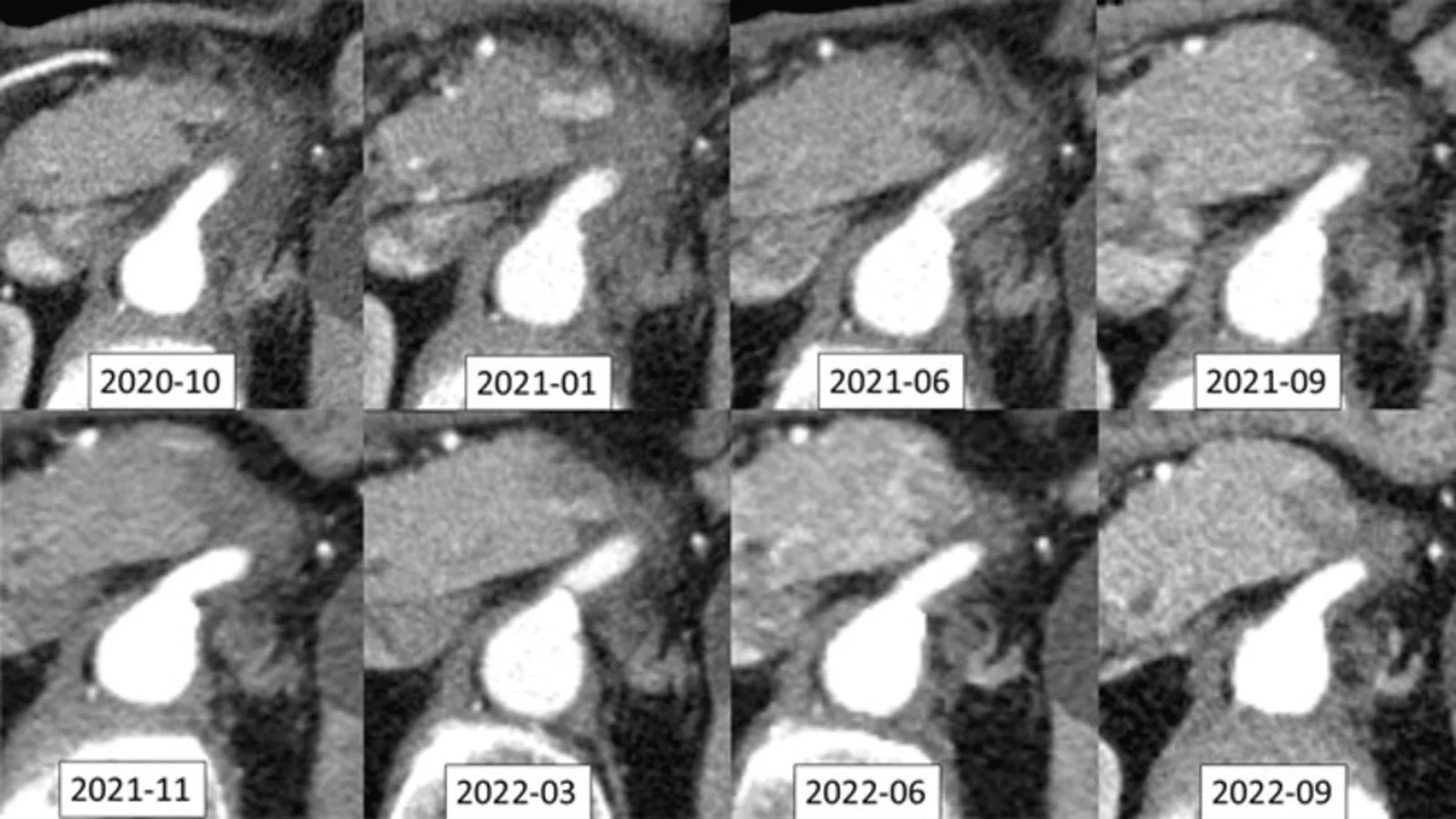
CT imaging of changes in primary tumor size and infiltration during liposomal irinotecan plus 5-FU treatment within proximity of the superior mesenteric artery 5-FU, fluorouracil; CT, computed tomography.

**Figure 6:**
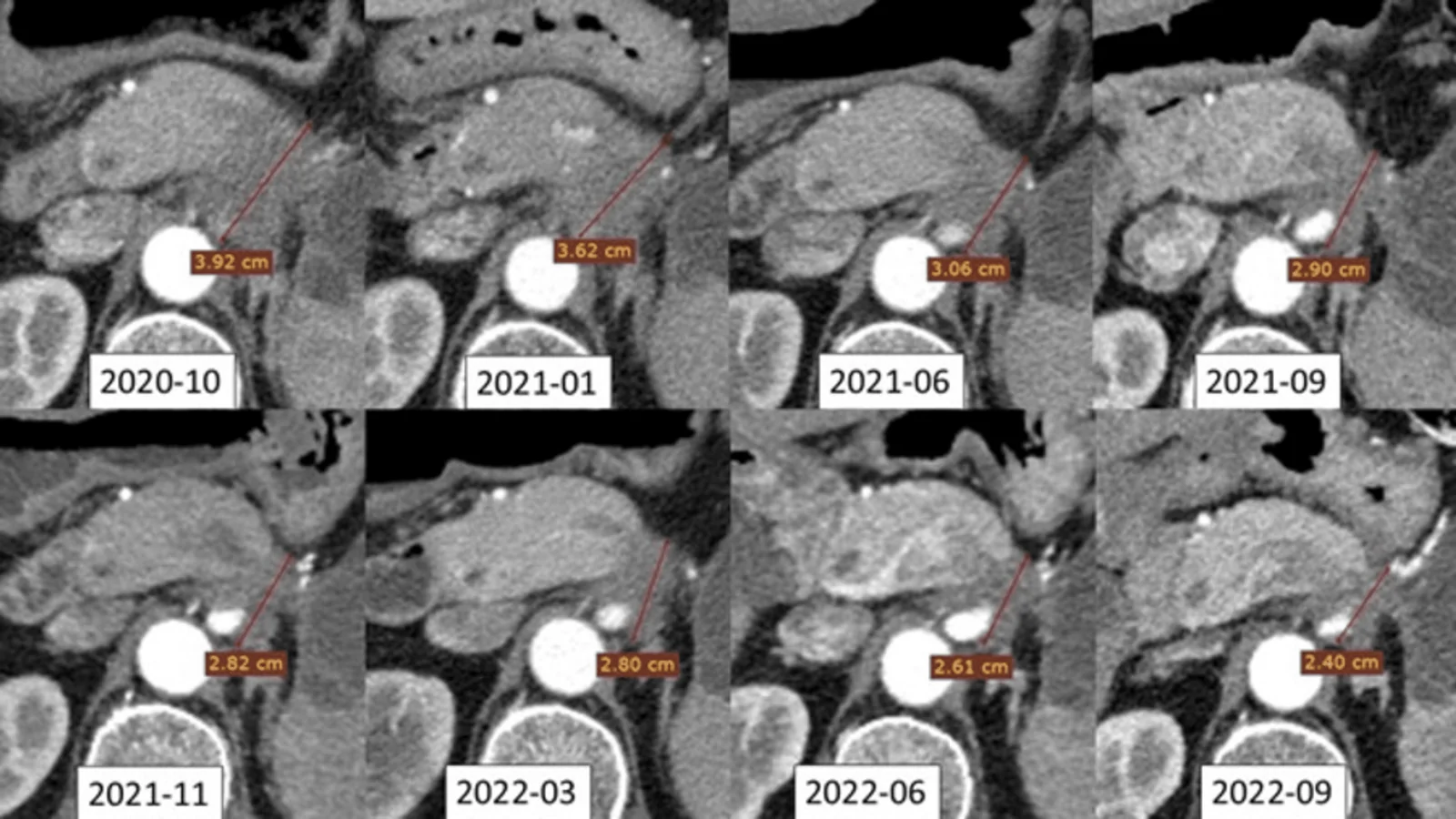
CT imaging of changes in primary tumor size and infiltration during liposomal irinotecan plus 5-FU treatment within proximity of the coeliac trunk 5-FU, fluorouracil; CT, computed tomography.

Two months after completing gemcitabine treatment (20 months after diagnosis), an abdominal CT scan revealed enlargement of the primary pancreatic tumor (diameter increase of approximately 5%), a pleural lesion, and the presence of new peritoneal carcinomatosis. Laboratory testing confirmed increased serum CA 19-9 (2395.2 kU/L; ULN 37 kU/L). The main symptoms reported by the patient at this time were pain and loss of appetite. As a consequence of the evident disease progression, third-line chemotherapy with liposomal irinotecan plus 5-FU/LV (liposomal irinotecan 70 mg/m², 5-FU 2400 mg/m², LV 400 mg/m²) was initiated a month later. Regular CT imaging revealed a reduction in tumor size and infiltration from 3.92 cm on the date of the post-second-line treatment CT scan to 2.40 cm 23 months later (Figure 5 and Figure 6). In the same month that reduction in tumor size and infiltration was observed, the patient tested positive for COVID-19 during chemotherapy but had no major COVID-related complications.

The patient continued with the liposomal irinotecan plus 5-FU/LV regimen for 35 months. During almost three years of treatment with liposomal irinotecan plus 5-FU/LV, the best response was PR (according to RECIST 1.1); the pleural lesion and peritoneal lesions decreased in diameter and eventually became undetectable, and CA 19-9 levels fluctuated but showed a general increasing trend and remained above the ULN. She had no significant AE but did experience CTCAE grade 2 diarrhea lasting approximately one month, occurring 28 months into treatment with liposomal irinotecan plus 5-FU/LV. Colonoscopy revealed no pathological changes, and the patient continued on liposomal irinotecan plus 5-FU/LV at a reduced dose (50% dose reduction of irinotecan and 25% dose reduction of 5-FU).

**Figure 7:**
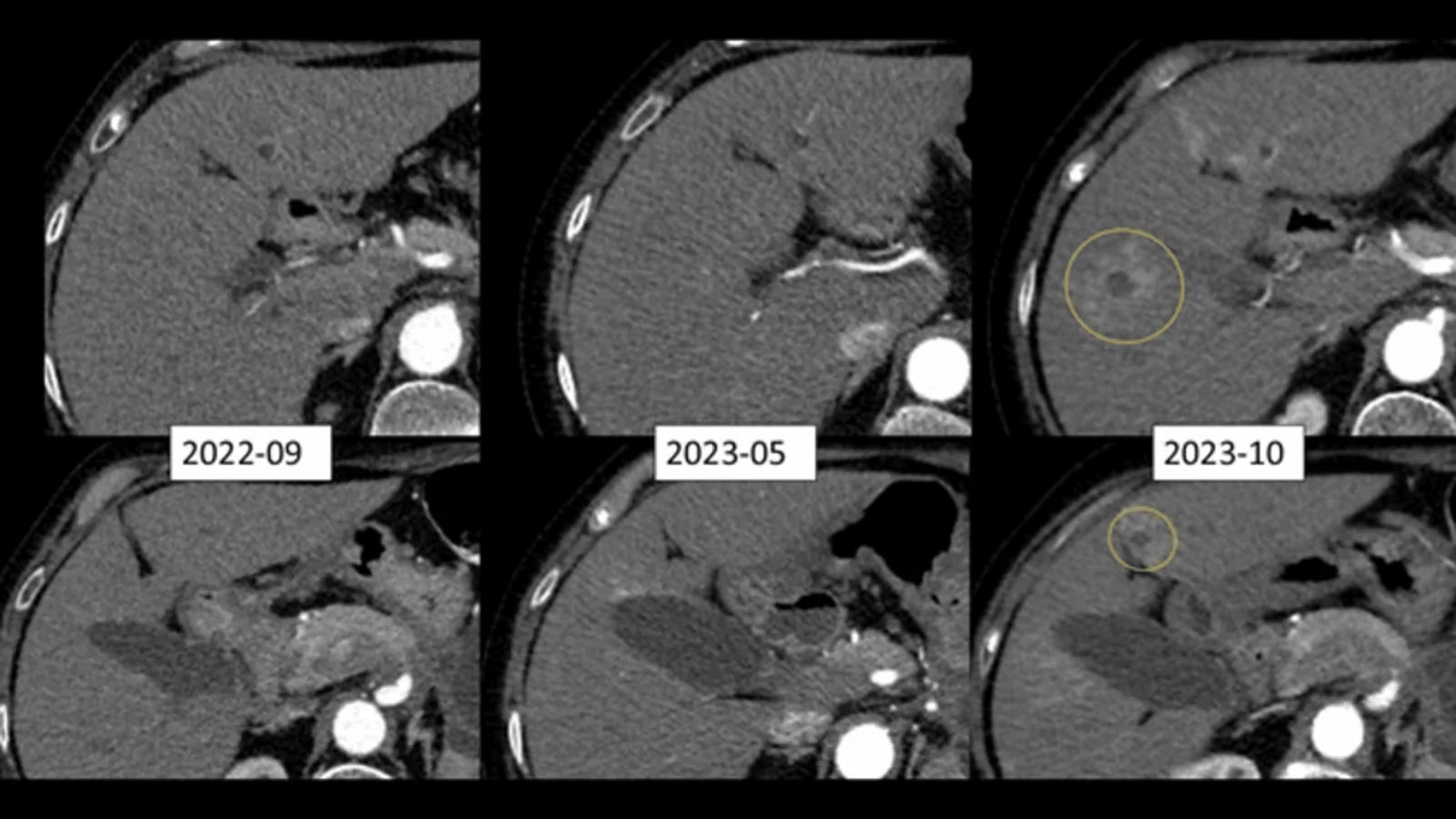
Serial CT imaging of the liver showing the development of metastatic lesions The yellow circle shows a metastatic lesion in the liver. CT, computed tomography.

A scheduled CT scan 56 months after diagnosis (when third-line treatment ended) identified new lesions in the liver (Figure 7) and resulted in a switch in chemotherapy to fourth-line cisplatin (25 mg/m²)/gemcitabine (1000 mg/m²) on days 1 and 8 of every 21-day cycle. As there is no standard-of-care fourth-line treatment regimen in Lithuanian guidelines, cisplatin + gemcitabine treatment was chosen because, throughout lines of treatment, the patient maintained a good functional status, with an ECOG PS score of 0. Five years after diagnosis (and four months after initiating fourth-line treatment), the patient continues treatment and has an ECOG PS score of 0.

Patient perspective

The patient, upon receiving the novel treatment, was pleased to note improvements in her everyday life. As this was her third-line treatment and she had previous experience with other regimens, she found this combination to be exceptionally well tolerated. Despite experiencing slight side effects initially, she demonstrated resilience by managing them effectively, requiring only minimal changes to her diet and daily routine.

The patient was familiar with the poor prognosis of pancreatic cancer, and this treatment increased hope for her. Even when the disease progressed on treatment, the patient's general condition remained quite good, and she could be treated further. It was essential to have the possibility to prolong her life with minimal side effects.

## Discussion

In this case report, we describe a female patient with locally advanced, unresectable PDAC who demonstrated a prolonged response during third-line treatment with liposomal irinotecan plus 5-FU/LV (nearly three years) and long-term survival (five years since diagnosis).

Approval of liposomal irinotecan plus 5-FU/LV for the treatment of metastatic PDAC in patients previously treated with gemcitabine-based therapy was based on evidence from the phase 3 NAPOLI-1 trial. Final OS analyses over an extended follow-up confirmed the approximately two-month survival advantage with the liposomal irinotecan combination compared with 5-FU/LV alone reported in the primary analysis [7,8]. Notably, 25% of patients receiving liposomal irinotecan plus 5-FU/LV in the study were alive for one year or more [7]. A systematic review of 21 studies found that the use of liposomal irinotecan plus 5-FU/LV resulted in a significantly longer OS compared to other second-line therapies [9]. This case compares favorably with outcomes in the NAPOLI-1 trial, with this patient experiencing sustained response with the liposomal irinotecan/5-FU/LV combination for almost three years, with the best response being PR. To our knowledge, this case represents the longest response to liposomal irinotecan combination therapy to date. Previous case studies in a 61-year-old Asian female patient and a 47-year-old African American male patient with advanced PDAC have reported prolonged response with combinations of liposomal irinotecan plus 5-FU and/or LV of 51 weeks and seven months, respectively [10,11].

The patient burden of pancreatic cancer is well established, with treatment side effects an acknowledged contributor to the reported decline in quality of life [12]. Encouragingly, treatments were well tolerated by the patient, and experienced side effects were infrequent (grade 3 thrombocytopenia with second-line gemcitabine and grade 2 diarrhea with liposomal irinotecan plus 5-FU/LV) and managed appropriately with dose reduction. Moreover, the patient demonstrated a good performance status throughout, suggesting minimal impact of treatment side effects on patient functioning.

The NAPOLI-1 study investigators conducted a post-hoc analysis to identify patient factors predisposing to long-term survival (one year or longer) [7]. The results showed that long-term survivors in the liposomal irinotecan plus 5-FU/LV group were more likely to be aged 65 years or younger; have a Karnofsky Performance Status score ≥90; a neutrophil-to-lymphocyte ratio of 5 or lower; a CA 19-9 level <59 × the ULN; and be less likely to have liver metastases than the overall liposomal irinotecan group. The patient described in our report fulfilled a number of criteria favoring long-term survival, including being aged below 65 years, good performance status (ECOG PS 0), and no liver metastases at treatment initiation. Of note, molecular profiling did not reveal any germline alterations that may provide clarification of the response status of our patient. It is possible that the exceptional response to liposomal irinotecan reported here could be due to somatic mutations not detected in the panel or to variants in DNA repair genes. Pathogenic alterations in the *ATM* gene were found to be associated with improved survival outcomes in a study of patients with pancreatic cancer, where patients with *ATM* gene alterations achieved a median OS that was 24.7 months longer than that of patients without *ATM* gene alterations [13]. In addition, genetic polymorphisms in cellular transporters, such as adenosine triphosphate-binding cassette transporters, which are involved in the metabolism and excretion of irinotecan, may result in pharmacokinetic variability between patients and impact responses to irinotecan [14]. Overexpression of the enzyme topoisomerase 1 occurs in around 52% of pancreatic cancer [15]. In a study of patients with metastatic colorectal cancer, progression-free survival was four months longer and OS was significantly longer (p = 0.0383) in those with topoisomerase 1 overexpression than in those without, which may be due to tumor cells having higher sensitivity to irinotecan [16]. There is a lack of data on overexpression of topoisomerase 1 in pancreatic cancer, and while the expression of topoisomerase 1 was not tested for in this case, overexpression could potentially contribute to the longer survival outcomes reported here. Overall, there was nothing remarkable about this patient’s disease or treatment course, which may limit the conclusions on why long-term survival was achieved in this patient specifically.

Limitations of this study include those universal to case reports, as this is a single patient; results are not generalizable to the wider population, and as treatment of this patient took place in a real-world setting, not all variables could be controlled for. For example, patient follow-up in this case may have been less regular than observed within the controlled environment of a clinical trial. In addition, data from genetic, laboratory, and clinical assessments/outcomes that would be collected in a clinical trial may not have been collected for this patient treated in the real-world. This is a limitation of this study, as an increased number of assessments/outcomes may have identified characteristics that could have contributed to the long-term survival demonstrated by this patient.

## Conclusions

This case study reports remarkable observations of a sustained response for almost three years with third-line liposomal irinotecan plus 5-FU/LV and long-term survival (five years since diagnosis) in our patient with locally advanced pancreatic cancer. This lends support to the prognostic value of delineating patient factors that positively impact outcome and therefore potentially alter the current bleak course of pancreatic cancer. As there were no remarkable features about this patient’s disease or treatment course, future research is required to further assess the patient factors identified here and others that may predict long-term survival in patients with locally advanced pancreatic cancer.
